# Development and validation of a nomogram for predicting COPD: A nationwide population-based study in South Korea

**DOI:** 10.1097/MD.0000000000039901

**Published:** 2024-09-27

**Authors:** Seungeun Oh, Hyungkyun Mok, Kyuhee Jo

**Affiliations:** a Seoul Women’s College of Nursing, Seoul, South Korea; b Department of Health Administration, Hanyang Women’s University, Seoul, South Korea; c College of Nursing, Korea University, Seoul, South Korea.

**Keywords:** COPD, KNHANSE, nomogram, spirometry

## Abstract

Chronic obstructive pulmonary disease (COPD) remains a significant global health burden exacerbated by tobacco smoking, occupational exposure, and air pollution. COPD is one of the top 3 causes of death worldwide. In South Korea, the COPD burden is expected to increase due to ongoing exposure to risk factors and the aging population. COPD is extensively underdiagnosed or underestimated, owing to a lack of public awareness. This study aimed to develop and validate a nomogram for COPD by using national data to promote early diagnosis and intervention. This study drew on a dataset from the 7th Korea National Health and Nutrition Examination Survey from 2016 to 2018, including 10,819 subjects aged 40 years or older with spirometry results. Influence of demographic, socioeconomic, and health-related factors on the incidence. Multivariable logistic regression was used to identify the significant predictors of the nomogram. The nomogram was validated using receiver operating characteristic curves, calibration plots, and concordance index (C-index). Internal validation was performed by bootstrapping. In the final analysis, 1059 (14.0%) participants had COPD. Key risk factors associated with increased COPD risk included being male, aged 70 and older, lower educational level, living in a rural area, current smoking status, underweight, and history of tuberculosis and asthma. The area under the curve (AUC) of the model was 0.822 (95% CI: 0.810–0.832), indicating that the nomogram has a high ability to identify COPD. The nomogram demonstrated solid predictive performance, as confirmed by calibration plots with a C-index (of 0.822) for the validation set with 1000 bootstrap samples. In conclusion, we developed a tool for the early detection of COPD with good properties in primary care settings, without spirometry. Appropriate and early diagnosis of COPD can have a crucial impact on public health.

## 1. Introduction

Global population aging and tobacco use, the primary risk factors for chronic obstructive pulmonary disease (COPD), contribute significantly to the increasing global burden of COPD.^[1,2]^ With 3.23 million deaths from COPD in 2019, COPD ranks as the third most common cause of mortality globally.^[3]^ In South Korea, COPD has been the top 10 cause of death for the past 20 years, with chronic lower respiratory diseases ranking as the ninth leading cause of death, accounting for 2.4% of all deaths in 2019.^[4]^ Despite the growing burden of COPD in South Korea, only 2.3% of people over the age of 40 are aware of the disease.^[5]^ As COPD is a disease whose treatment costs increase with severity, it is necessary to identify its risk factors and actively detect patients at an early stage to provide appropriate treatment.^[6]^ Smoking, occupational exposure, and air pollution are the primary causes and risk factors of COPD, a disease characterized by lung parenchymal damage and airway disease, which together limit lung function and produce various clinical symptoms.^[7]^ The prevalence of COPD is expected to continue to increase due to rising smoking rates among females, aging populations, and escalating risk factors such as air pollution, which negatively affect the quality of life of patients and their families and medical expenditure related to the exacerbation of respiratory symptoms.^[8]^ The prevalence of COPD in South Korea has increased since 2009. According to the 2019 National Health and Nutrition Examination Survey, 12.7% of adults aged 40 years and older were affected in 2019, with a higher prevalence among men (18.6%) than among women (7.1%) and an increase particularly noted among older adults.^[9]^ The increasing prevalence of COPD, which had an estimated global economic burden of approximately $4.326 trillion in 2017, is expected to significantly impact the global economy for years to come.^[10]^ In South Korea, the overall cost of COPD has increased; according to the Korea Health Insurance Review and Evaluation Service, direct medical expenses amounted to approximately $215 million in 2010, and by 2013, the total cost of COPD, including both direct and indirect costs, reached approximately $440 million.^[11,12]^

The socioeconomic burden of COPD in South Korea continues to increase.^[13]^ Therefore, it is essential to identify patients with a higher likelihood of developing COPD.^[14]^ A few studies have shown that efforts to predict COPD incidence using a nomogram in Korea either did not consider socioeconomic factors and smoking behaviors or were conducted in a single hospital.^[15,16]^

A nomogram is a visual representation of a multivariable model widely used in oncology for prognostic assessment.^[17,18]^ In this study, we identified independent risk factors using representative nationwide data from South Korea, and constructed a nomogram to estimate the probability of COPD.

## 2. Methods

### 2.1. Data source and study population

**Figure 1.**
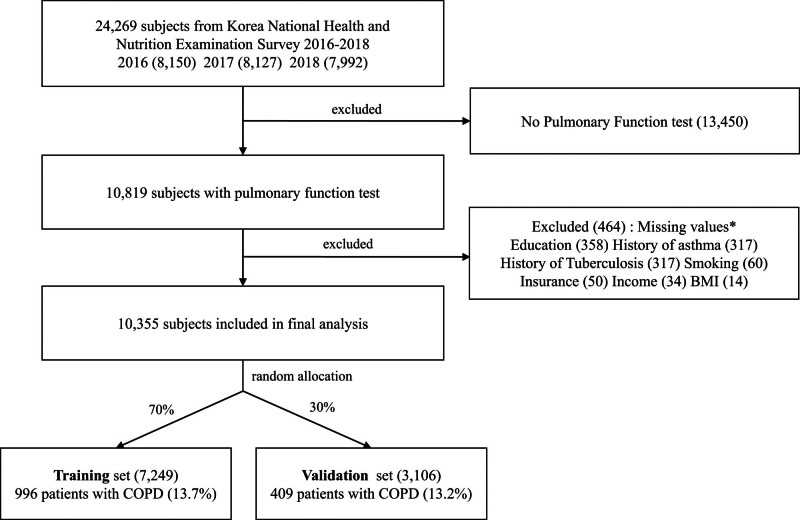
Study flow chart. BMI = body mass index, COPD = chronic obstructive pulmonary disease.

This study used data from the 7th Korea National Health and Nutrition Examination Survey (KNHANES) from 2016 to 2018. The KNHANES employs a stratified sampling method based on the South Korea Population and Housing Census, which has been conducted annually since 2007 to improve the health of the South Korean population. The study included 10,355 subjects aged 40 years or older, who were part of the 24,269 subjects who underwent pulmonary function tests with spirometry (Vyntus Spiro, Carefusion, Germany) conducted by trained nurses and technicians in the KNHANES during this period. Respondents with a forced expiratory volume in 1 second to forced vital capacity ratio of <70% were classified as living with COPD according to the Korean Academy of Tuberculosis and Respiratory Diseases and the Global Initiative for Chronic Obstructive Lung Disease.^[7,14]^ We excluded subjects under 40 years of age and those with missing values. Some respondents had multiple missing variables. The selection process was detailed in Figure 1.

### 2.2. Covariate

The study adjusted for 12 variables: sex, age, education, income, marital status, residence area, national health insurance (NHI) program, smoking status, history of asthma, history of tuberculosis (TB), and body mass index (BMI). Age was categorized into 4 groups: 40 to 49, 50 to 59, 60 to 69, and more 70 years. Education level was divided into 2 categories: middle school or less and high school or more. Income was categorized into quintiles, from the highest 20% to the lowest 20%, based on sex and age-specific income distribution. Marital status was categorized as married or cohabiting, divorced or widowed, or never married. The residence area was defined as either urban or rural. Based on individuals’ addresses, urban areas were defined as administrative units at the “Dong” level or above, while rural areas referred to nonurban areas, such as “Eup” or “Myeon.” The NHI program was divided into NHI and medical aid programs. Smoking status was classified as never smoked, former smokers, or current smokers. Current smokers were those who reported smoking daily or occasionally, and had consumed more than 100 cigarettes in their lives. Former smokers were defined as individuals who had smoked regularly but no longer smoked daily and had consumed more than 100 cigarettes. Never smokers were those who either reported never smoking or had smoked fewer than 100 cigarettes in their lives. A history of asthma and TB represents whether an individual has ever been diagnosed with asthma or TB by a physician in their lifetime. BMI (kg/m^2^) was categorized as underweight if below 18.5, normal between 18.5 and 23, and overweight or obese if >23.

### 2.3. Statistical analysis

To construct and validate the nomogram, all cases were randomly split into a training set (n = 7249) and a validation set (n = 3106) in a 7:3 ratio. Categorical variables were described as numbers and percentages (%), and the *χ*^2^-test used for comparison. Univariate logistic regression was performed to analyze the relationship between each risk factor and COPD. We conducted multivariable logistic regression to identify independent predictors of COPD. The Hosmer–Lemeshow test was used to assess the goodness-of-fit of the model. We developed a nomogram from the training set to estimate the probability of an individual developing COPD. The efficacy of the nomogram was tested by discrimination and calibration.^[15]^ The performance of the nomogram was evaluated using the concordance index (C-index), receiver operating characteristic curves, and calibration plots with 1000 bootstrap resamples to reduce overfitting. The C-index was utilized to measure the ability to discriminate among different outcomes, with a higher C-index value corresponding to superior model performance. Calibration plots were used to assess the agreement between predicted and observed probabilities. All statistical analyses were performed with R software (version 4.4.0, R Foundation, https://www.r-project.org/), and a *P*-value of <.05 was considered statistically significant.

### 2.4. Ethical consideration

The ethical aspects of this study, including the use of data, study plan, and research ethics pledge, were approved by the Institutional Review Board of the Korea National Institute for Bioethics Policy (approval number: P01-202401-008).

## 3. Results

### 3.1. General characteristics

The total number of respondents was 10,355, including 4522 (43.7%) males and 5833 (56.3%) females. The subjects were divided into a training set (n = 7249) and validation set (n = 3106). In the training set, 2098 (28.9%) were aged 50 to 59 years, 4492 (62.0%) were high school graduates or higher, and 1211 (16.7%) were current smokers. In addition, 996 (13.7%) had living with COPD. The characteristics of the participants in the training and validation sets were similar, with no statistically significant differences observed between the 2 groups for most variables (*P* > .05), except for marital status (*P* = .045). The results were presented in Table 1.

**Table 1:** Demographic and clinical characteristics of the study cohort.

Variable		Training set (n = 7249)		Validation set (n = 3106)			Overall (n = 10355)		*x* ^2^	*P*-value
		N	%	N	%	N		%		
Sex	Male	3169	43.7	1353	43.6	4522		43.7	0.021	.884
	Female	4080	56.3	1753	56.4	5833		56.3		
Age (years)	40–49	1954	27.0	865	27.9	2819		27.2	1.601	.659
	50–59	2098	28.9	869	28.0	2967		28.6		
	60–69	1794	24.7	781	25.1	2575		24.9		
	70+	1403	19.4	591	19.0	1994		19.3		
Education level	Middle school or less	2757	38.0	1183	38.1	3940		38.0	0.003	.958
	High school or more	4492	62.0	1923	61.9	6415		62.0		
Income (Quintiles)	Q1 (lowest 20%)	1306	18.0	610	19.6	1916		18.5	7.320	.120
	Q2	1422	19.6	621	20.0	2043		19.7		
	Q3	1436	19.9	635	20.5	2071		20.0		
	Q4	1554	21.4	622	20.0	2176		21.0		
	Q5 (highest 20%)	1531	21.1	618	19.9	2149		20.8		
Marital status	Married	5847	80.7	2486	80.1	8333		80.5	6.209	.045
	Single (never married)	210	2.9	119	3.8	329		3.2		
	Divorced, widowed, separated, missing	1192	16.4	501	16.1	1693		16.3		
Region	Urban area	5815	80.2	2511	80.8	8326		80.4	0.540	.462
	Rural area	1434	19.8	595	19.2	2029		19.6		
National Health Insurance Program	NHI	6986	96.4	2985	96.1	9971		96.3	0.436	.509
	Medical aid	263	3.6	121	3.9	384		3.7		
Smoking status	Never-smoker	4275	59.0	1867	60.1	6142		59.3	1.168	.558
	Ex-smoker	1763	24.3	733	23.6	2496		24.1		
	Current-smoker	1211	16.7	506	16.3	1717		16.6		
Asthma	No	7056	97.3	3012	97.0	10,068		97.2	1.069	.301
	Yes	193	2.7	94	3.0	287		2.8		
TB	No	6903	95.2	2960	95.3	9863		95.2	0.025	.874
	Yes	346	4.8	146	4.7	492		4.8		
BMI	<18.5	136	1.9	61	2.0	197		1.9	0.129	.937
	18.5–23	5294	73.0	2272	73.1	7566		73.1		
	≥23	1819	25.1	773	24.9	2592		25.0		
COPD	No	6253	86.3	2697	86.8	8950		86.4	0.606	.436
	Yes	996	13.7	409	13.2	1405		13.6		

BMI = body mass index, COPD = chronic obstructive pulmonary disease, NHI = national health insurance, TB = tuberculosis.

### 3.2. Demographics and clinical characteristics comparison by COPD groups

All study variables showed statistically significant differences (*P*-value < .05) between the COPD and non-COPD groups. The number of COPD cases increased with age, and 719 (72.2%) COPD were male. The educational level was lower in the COPD group, with 552 (55.4%) having a middle school education or less, compared to 2205 (35.3%) in the non-COPD group. Current and ex-smokers were more prevalent in the COPD group (27.7% and 40.9%, respectively) than in the non-COPD group (15.0% and 21.7%, respectively). COPD also had higher rates of asthma (8.0% vs 1.8%) and a history of TB (11.4% vs 3.7%). Details are shown in Table 2.

**Table 2:** Demographic and clinical characteristics by non-COPD and COPD groups in training set.

Variable		Non-COPD (n = 6253)		COPD (n = 996)		Overall (n = 7249)		*x* ^2^	*P*-value
		N	%	N	%	N	%		
Sex	Male	2450	39.2	719	72.2	3169	43.7	380.426	<.001
	Female	3803	60.8	277	27.8	4080	56.3		
Age (years)	40–49	1890	30.2	64	6.4	1954	27.0	600.035	<.001
	50–59	1926	30.8	172	17.3	2098	28.9		
	60–69	1461	23.4	333	33.4	1794	24.7		
	70+	976	15.6	427	42.9	1403	19.4		
Education level	Middle school or less	2205	35.3	552	55.4	2757	38.0	148.139	<.001
	High school or more	4048	64.7	444	44.6	4492	62.0		
Income (quintiles)	Q1 (lowest 20%)	1091	17.4	215	21.5	1306	18.0	14.174	<.01
	Q2	1217	19.5	205	20.6	1422	19.6		
	Q3	1264	20.2	172	17.3	1436	19.9		
	Q4	1343	21.5	211	21.2	1554	21.4		
	Q5 (highest 20%)	1338	21.4	193	19.4	1531	21.1		
Marital status	Married	5052	80.8	795	79.8	5847	80.7	11.307	<.01
	Single (never married)	195	3.1	15	1.5	210	2.9		
	Divorced, widowed, separated, missing	1006	16.1	186	18.7	1192	16.4		
Region	Urban area	5092	81.4	723	72.6	5815	80.2	42.333	<.001
	Rural area	1161	18.6	273	27.4	1434	19.8		
National health insurance program	NHI	6046	96.7	940	94.4	6986	96.4	13.136	<.001
	Medical aid	207	3.3	56	5.6	263	3.6		
Smoking status	Never-smoker	3962	63.3	313	31.4	4275	59.0	362.218	<.001
	Ex-smoker	1356	21.7	407	40.9	1763	24.3		
	Current-smoker	935	15.0	276	27.7	1211	16.7		
Asthma	No	6140	98.2	916	92.0	7056	97.3	128.466	<.001
	Yes	113	1.8	80	8.0	193	2.7		
TB	No	6021	96.3	882	88.6	6903	95.2	113.109	<.001
	Yes	232	3.7	114	11.4	346	4.8		
BMI	<18.5	108	1.7	28	2.8	136	1.9	19.573	<.001
	18.5–23	4526	72.4	768	77.1	5294	73.0		
	≥23	1619	25.9	200	20.1	1819	25.1		

BMI = body mass index, COPD = chronic obstructive pulmonary disease, NHI = national health insurance, TB = tuberculosis.

### 3.3. Predictors of COPD

In the logistic regression analysis, age [40–49 reference group vs 70+: univariable OR: 12.920 (9.825–16.990), multivariable OR: 14.085 (10.226–19.399)], history of asthma in lifetime [no reference group vs yes: univariable OR: 4.746 (3.534–6.372), multivariable OR: 5.204 (3.665–7.390)], smoking status [never-smoker reference group vs current-smoker: univariable OR: 3.799 (3.238–4.457), multivariable OR: 3.338 (2.573–4.332)], and sex [female reference group vs male: univariable OR: 4.029 (3.476–4.670), multivariable OR: 2.811 (2.197–3.597)] were strongly associated with COPD risk. Education level [high school or more reference group vs middle school or less: univariable OR: 2.282 (1.994–2.613), multivariable OR: 1.241 (1.038–1.485)] and BMI [≥23 reference group vs <18.5: univariable OR: 2.099 (1.351–3.261), multivariable OR: 2.069 (1.204–3.557)] remained significant factors in both analyses. In the Health insurance program, NHI beneficiaries showed a significant association with medical aid program recipients in the univariable analysis (OR: 1.740 [1.285–2.356], *P* < .001), but not in the multivariable analysis (OR: 1.086 [0.756–1.561], *P* = .655). Regarding marital status, being married compared to being single (never married) showed a significant association in the univariable analysis (OR: 2.046 [1.204–3.477], *P* = .008), but not in the multivariable analysis (OR: 1.350 [0.755–2.415], *P* = .311). Likewise, being divorced, widowed, separated, or missing demonstrated a significant association in the univariable analysis (OR: 2.404 [1.390–4.158], *P* = .002), but not in the multivariable analysis (OR: 1.392 [0.763–2.540], *P* = .281). Marital status and the national health insurance program were significant in the univariable analysis but not in the multivariable analysis. The Hosmer–Lemeshow test indicated a good fit for the model (*P* = .288). These results suggest that older age, male sex, lower educational level, smoking status, diagnosis of asthma and TB, and lower body mass index are significant risk factors for COPD. The results are presented in Table 3.

**Table 3:** Multivariable logistic regression in the training set.

	Univariable		Multivariable	
	OR (95% CI)	*P*-value	OR (95% CI)	*P*-value
**Sex**				
Female (ref)				
Male	4.029 (3.476–4.670)	<.001	2.811 (2.197–3.597)	<.001
**Age**				
40–49 (ref)				
50–59	2.637 (1.966–3.538)	<.001	2.745 (2.019–3.732)	<.001
60–69	6.731 (5.107–8.871)	<.001	7.052 (5.194–9.574)	<.001
70+	12.920 (9.825–16.990)	<.001	14.085 (10.226–19.399)	<.001
**Education level**				
High school or more (ref)				
Middle school or less	2.282 (1.994–2.613)	<.001	1.241 (1.038–1.485)	.018
**Income (Quintiles**)				
Q5 (highest 20%) (ref)				
Q4	1.089 (0.883–1.343)	.424	1.057 (0.837–1.335)	.641
Q3	0.943 (0.758–1.175)	.603	0.857 (0.669–1.099)	.223
Q2	1.168 (0.945–1.443)	.150	1.119 (0.878–1.427)	.363
Q1 (lowest 20%)	1.366 (1.107–1.686)	.004	1.158 (0.900–1.489)	.254
**Marital status**				
Single (never married) (ref)				
Married	2.046 (1.204–3.477)	.008	1.350 (0.755–2.415)	.311
Divorced, Widowed, Separated, Missing	2.404 (1.390–4.158)	.002	1.392 (0.763–2.540)	.281
**Region**				
Urban area (ref)				
Rural area	1.656 (1.421–1.930)	<.001	1.198 (1.006–1.428)	.043
**National Health Insurance program**				
NHI (ref)				
Medical aid	1.740 (1.285–2.356)	<.001	1.086 (0.756–1.561)	.655
**Smoking status**				
Never-smoker (ref)				
Ex-smoker	3.737 (3.131–4.459)	<.001	1.928 (1.510–2.460)	<.001
Current-smoker	3.799 (3.238–4.457)	<.001	3.338 (2.573–4.332)	<.001
**Asthma**				
No (ref)				
Yes	4.746 (3.534–6.372)	<.001	5.204 (3.665–7.390)	<.001
**TB**				
No (ref)				
Yes	3.354 (2.652–4.243)	<.001	2.505 (1.909–3.288)	<.001
**BMI**				
≥23 (ref)				
18.5–23	1.374 (1.164–1.621)	<.001	1.503 (1.252–1.803)	<.001
<18.5	2.099 (1.351–3.261)	.001	2.069 (1.204–3.557)	.009

BMI = body mass index, NHI = national health insurance, TB = tuberculosis.

### 3.4. Nomogram predicting the probability of developing COPD

**Figure 2.**
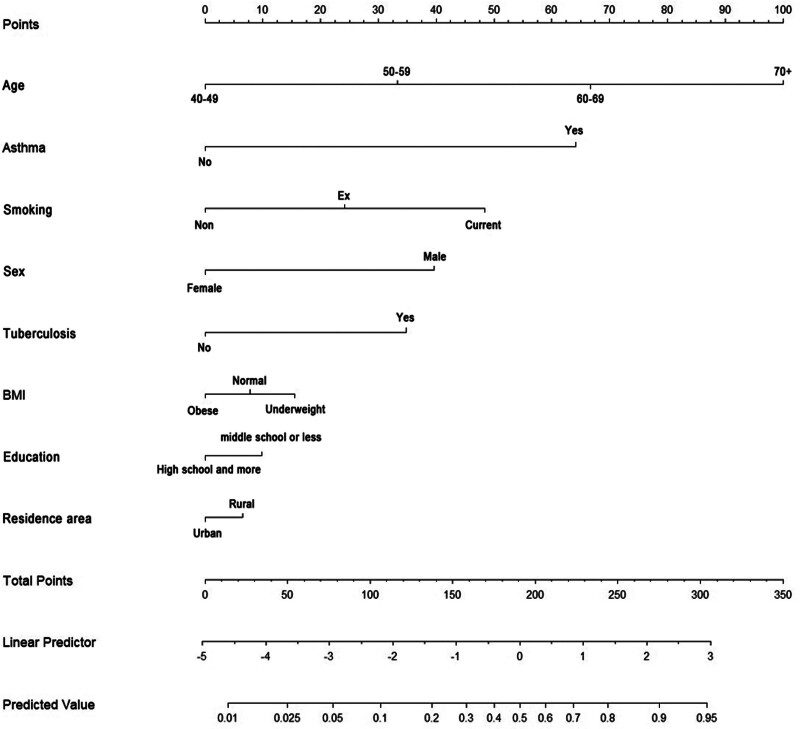
Nomogram for predicting COPD. BMI = body mass index, COPD = chronic obstructive pulmonary disease.

**Figure 3.**
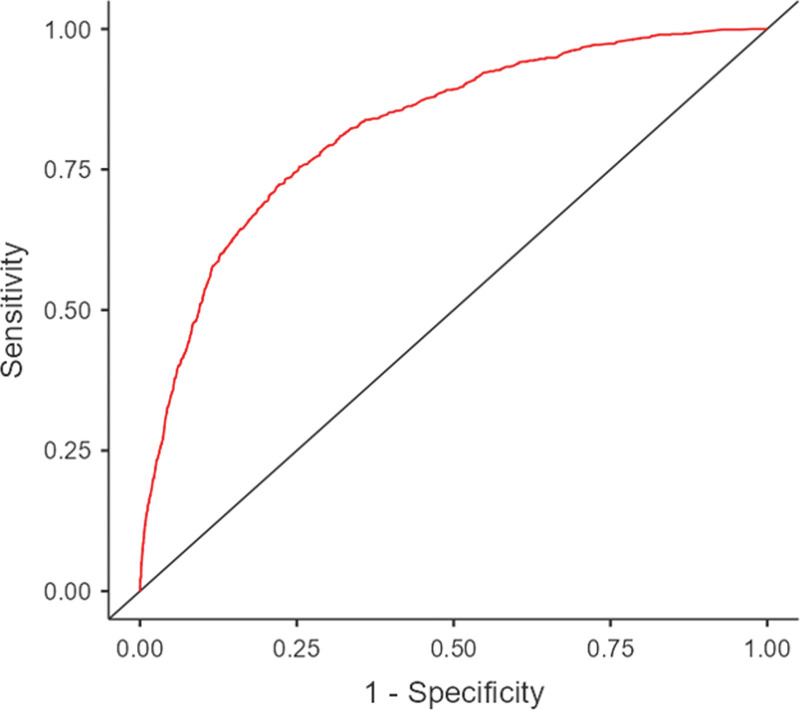
ROC curve for nomogram for predicting the risk of COPD. COPD = chronic obstructive pulmonary disease, ROC = receiving operating characteristics.

**Figure 4.**
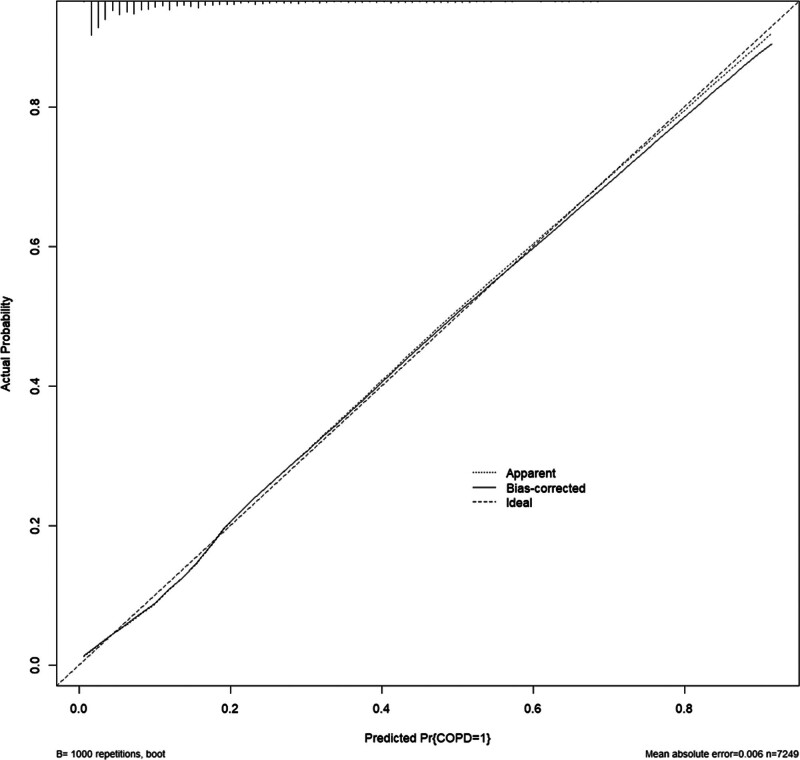
Internal validation of nomogram using bootstrap sampling (1000 repetitions).

Based on the multivariable logistic regression analysis, we constructed a nomogram to predict the probability of COPD incidence, as shown in Figure 2. The nomogram showed 8 significant predictors: age, sex, smoking status, asthma history, TB history, BMI, education level, and residence area. The logistic regression model underlying the nomogram showed strong statistical significance (*P* < .001) with an *R*^2^ of 0.292, indicating a good overall fit. All included predictors, except for income, marital status, NHI, and residential area, were statistically significant (*P* < .05) in this model. In the nomogram, draw a vertical line from each variable to the “Points” line at the top. The points for all variables are summed to obtain a total point score, which is then located on the ``Total Points’’ line. The vertical line drawn from this point to the predicted value line indicates the probability of COPD. We further validated the performance of the nomogram using the concordance index (C-index) to suggest a model discrimination. In the training set, the C-index was 0.822, which displayed good model discrimination between the predicted probabilities and actual outcomes. The receiver operating characteristic curve calculated area under the curve (AUC) was 0.822 (95% CI: 0.810–0.832); suggesting good model accuracy, as shown in Figure 3. The calibration plot in Figure 4 demonstrated the nomogram for predicting COPD in the training set was close to the ideal curve. Figure 4 showed good correlation and strong calibration in predicting the probability of COPD incidence. The model’s performance was validated through internal sampling using the bootstrap method.

## 4. Discussion

Our study revealed that being male, aged 70 years and older, having lower education, living in rural areas, current cigarette smoking, being underweight, and having a history of TB or asthma were significantly associated with COPD incidence. The rising prevalence and exacerbation of COPD in South Korea have led to its recognition as a crucial public health issue. Effective management is important for early detection, proper intervention, and improvement of quality of life by minimizing exacerbation.^[19]^ The World Health Organization has recognized the significance of COPD.^[20]^ By analyzing key risk factors based on demographic, socioeconomic status, and health-related variables, healthcare providers can quickly screen patients at the primary care level, allowing governments to potentially reduce the burden associated with COPD. Recent studies have indicated that the incidence of COPD becomes more common as individuals age,^[21–23]^ and lung function gradually decreases over time.^[24,25]^ These factors contribute to the likelihood of developing COPD in older adults. It highlights the vitality of developing strategies for early detection, prevention, and optimal management. Tobacco smoking is a significant risk factor for COPD. Our results indicate that current smokers have a higher risk of COPD,^[26–28]^ and smoking cessation has been identified as one of the most effective approaches to prevent the exacerbation of COPD.^[29,30]^ Consequently, anti-smoking initiatives and sustained tobacco control measures are essential, with a particular focus on younger age groups and women.^[31]^ Our study demonstrates a relationship between chronic respiratory conditions and COPD. Research participants with a history of TB diagnosis have a higher likelihood of developing COPD due to impaired lung function.^[32]^ Recent studies have also identified TB as a risk factor contributing to COPD,^[33,34]^ with asthma being acknowledged as another factor that increases this risk.^[35]^ Our finding that men had a higher likelihood of developing COPD than women is consistent with those of previous studies.^[36,37]^ Recent research indicates an increasing smoking rate among young Asian women, as well as an increase in the probability of smoking-related chronic diseases such as COPD.^[38]^ The smoking prevalence among young women in South Korea has been increasing because of the increasing use of e-cigarettes and heated tobacco products.^[39,40]^ It is anticipated that specific preventative and management strategies targeting women are necessary to address these changes. Recent studies have shown that low body mass is associated with worse outcomes in people with COPD.^[41,42]^ Low body weight is also associated with decreased lung function, which can be the likelihood of COPD,^[43,44]^ and emphasizes the maintenance of a healthy weight in individuals. COPD is associated with the educational level. Education has a direct relationship with the self-management of COPD because socioeconomic factors play a significant role in the development of the disease.^[45]^ People with higher educational levels are more likely to recognize smoking risk factors, and they are also more likely to maintain a healthy lifestyle and habits than those with lower educational levels^[46,47]^ Geographical location could influence the higher prevalence of COPD. Living in rural areas may increase the occupational exposure. The inequity in clinical outcomes related to COPD might be due to factors such as lower socioeconomic status, medical accessibility, and uninsured rates.^[48–50]^ There is an urgent need for preventive policies to address these unmet healthcare needs for early diagnosis of COPD.^[51]^ This study had several limitations. First, it is possible that smoking-related information may have had a recall bias due to poor memory from a long time ago by self-reported. Second, there is a lack of detailed data on the respondents’ smoking period and amount. Third, concerns exist regarding the applicability of our research results to countries with socioeconomic levels comparable to those in Korea. Future research should investigate the use of more advanced methods to accurately collect smoking information and generalize the results to a larger population. It is also important to develop practical applications of research findings, such as using nomograms and targeted prevention programs for high-risk groups, to ensure that research results are effectively promoted for public health.

We analyzed the risk factors for COPD in South Korea using representative national data from KNHANES from 2016 to 2018. We found that males, 70+ years old, middle school or less, living in a rural area, current smokers, underweight, TB, and asthma were associated with an increased risk of COPD. We constructed and validated a nomogram to predict COPD, which may help healthcare providers achieve early detection and diagnosis in a primary setting without spirometry. This nomogram could reduce the burden on national healthcare systems through early diagnosis and identification of high-risk individuals.

## 5. Conclusion

A nationwide population-based study in South Korea identified the potential risk factors for developing COPD. We found that males, age 70 years and above, middle school education or less, living in a rural area, current smoking status, underweight, a history of TB, and asthma were associated with COPD. We developed a predictive model presented as a nomogram—a tool that healthcare providers can use to make decisions regarding the early screening and prevention of COPD in the community. We recommend applying nomograms and developing preventive programs tailored to distinct risk groups. We highly recommend further research to thoroughly validate the tool and evaluate the risk associated with other factors, such as socioeconomic status and complex smoking behaviors.

## Author contributions

**Conceptualization:** Seungeun Oh.

**Data curation:** Kyuhee Jo.

**Formal analysis:** Seungeun Oh.

**Methodology:** Hyungkyun Mok.

**Writing – original draft:** Seungeun Oh.

**Writing – review & editing:** Kyuhee Jo.
